# Gastralgia

**Published:** 1901-10-12

**Authors:** 


### GASTRALGIA.

Professor Saundby[1] divides cases of gastralgia into two groups, one in which the pain occurs independently of eating and is relieved by a sufficient meal, or a small quantity of alcoholic stimulant; and the other in which the pain occurs after a meal and is distinctly to be referred to the introduction of food into the stomach. Cases in the first group are characteristic and typical. They occur in men and women of the neurasthenic type; perhaps more frequently in men than in women; usually in thin ardent excitable young persons, strenuous in the pursuit of work or pleasure, who exhibit in other respects the stigmata of neurasthenia. The pain comes from fatigue, exhaustion, excitement, emotion, or going too long without food; it is a gnawing, weary aching at the pit of the stomach, and is accompanied by mental depression, irritability of temper, and incapacity for work. This condition is generally called dyspepsia, not only by the patient but even by his medical adviser, although the patient may have his suspicions as to the correctness of the diagnosis on account of the relief he finds from a good meal. The rational treatment of such a condition is that of neurasthenia—rest, a Weir-Mitchell course, change of scene, a sea voyage or mountain air, with abundant food at regular intervals; and on recovery he must be warned against fatigue and the abuse of those nerve-destroyers tea and tobacco. If in the meantime it is necessary to use palliatives, benefit may be derived from iron, quinine, arsenic, nux vomica, and mineral acids, while the pain is relieved by small doses ($\frac{1}{16}$ to $\frac{1}{8}$ of a grain) of morphine or heroine hydrochloride in a teaspoonful of water. If this dose succeeds all well and good, but it should not be increased. If it does not relieve something else should be tried.

The pain that comes on after food is more likely to be confused with organic disease. In anæmic girls, for example, where pain directly follows the swallowing of anything, it may be very difficult to differentiate the condition from ulcer of the stomach, but in the purely nervous cases the pain is apt to be more constant and less dependent upon the quality or quantity swallowed. Vomiting with consequent relief of pain takes place in both cases, but in gastralgia there is no hæmatemasis, although there may be a little blood from the gums or pharynx. The treatment is bed, and milk and lime water in small quantities, such quantities, in fact, as the stomach will bear without pain; and if it will not bear them, then nutrient enemata every four hours. As a rule bed at once calms the pain. As soon as tolerance is established the quantity of milk should be rapidly increased, and as soon as they can be borne other things should be added or substituted, and in anæmic cases iron and sulphate of magnesia may also be given. The bowels should be kept open by a dose of Rubinat water every morning and massage is very desirable while resting in bed. It must be remembered that cases of organic disease of the stomach may be associated with a good deal of gastralgia. In such cases one has not to do with a pure neurosis, and it may be difficult to say how much of the pain is or is not of nervous origin. Of course in all such cases a careful examination of the stomach should be made, both as to its chemical and motor functions. Nor must we confine our attention to the stomach. We must look to the teeth, to the mastication, and to the quantity and quality of the food, to the general surroundings, and to checking the abuse of tea and tobacco, while bismuth and soda may be given before each meal, and the bowels be kept regular by an early morning dose of Carlsbad salts or Rubinat water.

[1] Treatment, Sep. 1901.

